# Adjuvant chemoradiotherapy of advanced resectable rectal cancer: results of a randomised trial comparing modulation of 5-fluorouracil with folinic acid or with interferon-*α*

**DOI:** 10.1038/sj.bjc.6605871

**Published:** 2010-09-28

**Authors:** M Kornmann, L Staib, T Wiegel, E-D Kreuser, M Kron, W Baumann, D Henne-Bruns, K-H Link

**Affiliations:** 1 Department of General, Visceral, and Transplantation Surgery, University of Ulm, Steinhoevelstrasse, Ulm 89075, Germany; 2 Study Group Oncology of Gastrointestinal Tumors (FOGT), Wiesbaden 65197, Germany; 3 Department of Radiation Oncology, University of Ulm, Ulm 89075, Germany; 4 Institute of Biometrics, University of Ulm, Ulm 89075, Germany

**Keywords:** rectal cancer, adjuvant chemoradiotherapy, 5-fluorouracil, interferon, folinic acid

## Abstract

**Background:**

Standard adjuvant chemoradiotherapy of rectal cancer still consists of 5-fluorouracil (5-FU) only. Its cytotoxicity is enhanced by folinic acid (FA) and interferon-*α* (INF*α*). In this trial, the effects of FA and IFN*α* on adjuvant 5-FU chemoradiotherapy in locally advanced rectal cancer were investigated.

**Methods:**

Patients with *R*_0_-resected rectal cancer (UICC stage II and III) were stratified and randomised to a 12-month adjuvant chemoradiotherapy with 5-FU, 5-FU+FA, or 5-FU+IFN*α*. All patients received levamisol and local irradiation with 50.4 Gy.

**Results:**

Median follow-up was 4.9 years (*n*=796). Toxicities (WHO III+IV) were observed in 32, 28, and 58% of patients receiving 5-FU, 5-FU+FA, and 5-FU+IFN*α*, respectively. No differences between the groups were observed for local or distant recurrence. Five-year overall survival (OS) rates were 60.3% (95% confidence interval (CI): 54.3–65.8), 60.4% (54.4–65.8), and 59.9% (53.0–66.1) for 5-FU, 5-FU+FA, and 5-FU+IFN*α*, respectively. A subgroup analysis in stage II (pT3/4pN0) disease (*n*=271) revealed that the addition of FA tended to reduce the 5-year local recurrence (LR) rate by 55% and increase recurrence-free survival and OS rates by 12 and 13%, respectively, relative to 5-FU alone.

**Conclusions:**

Interferon-*α* cannot be recommended for adjuvant chemoradiotherapy of rectal cancer. In UICC stage II disease, the addition of FA tended to lower LR and increased survival. The addition of FA to 5-FU may be an effective option for adjuvant chemoradiotherapy of UICC stage II rectal cancer.

During the last two decades, treatment strategies of rectal cancer have improved markedly. Although in the early 1990s local recurrence (LR) rates beyond 20% and overall recurrence rates beyond 50% were reported for UICC stage II and III (Gastrointestinal Tumor Study Group, 1985; Fisher *et al*, 1988; Krook *et al*, 1991), multimodal approaches were shown to increase local control and survival (Swedish Rectal Cancer Trial, 1997; Wolmark *et al*, 2000). In parallel, total mesorectal excision (TME), including the complete removal of the fatty tissue and lymph nodes surrounding the rectum, was introduced resulting in a significant improvement of local control (Martling *et al*, 2000; Wibe *et al*, 2002). Local recurrence rates were further decreased in locally advanced rectal cancer using neoadjuvant strategies compared with the adjuvant setting (Sauer *et al*, 2004) or combining radiation with chemotherapy (Bosset *et al*, 2006; Gérard *et al*, 2006). In contrast to the old resection technique (Swedish Rectal Cancer Trial, 1997), the addition of neoadjuvant radiation to modern TME surgery reduced LR, but did not improve survival (Peeters *et al*, 2007).

Irrespective of pre- or postoperative (chemo)radiation, distant metastases still occur in about 40% of locally advanced rectal cancers (Swedish Rectal Cancer Trial, 1997; Wolmark *et al*, 2000; Tepper *et al*, 2002; Sauer *et al*, 2004; Peeters *et al*, 2007). In order to improve prognosis, systemic treatment of these patients has to be optimised (Weiss *et al*, 2009). Marked advances in adjuvant treatment have been achieved in colon cancer during the last two decades (IMPACT investigators, 1995; Porschen *et al*, 2001; Haller *et al*, 2005; Link *et al*, 2005; Kuebler *et al*, 2007; André *et al*, 2009). Despite the clear benefit of 5-fluorouracil (5-FU) modulation by folinic acid (FA) in colon cancer (IMPACT investigators, 1995; Porschen *et al*, 2001; Haller *et al*, 2005; Link *et al*, 2005), a clear benefit of this combination in rectal cancer could not be shown (QUASAR Collaborative Group, 2000; Wolmark *et al*, 2000; Tepper *et al*, 2002; Dahl *et al*, 2009). Standard chemoradiotherapy of rectal cancer (UICC stage II and III) is often still carried out using 5-FU monotherapy (de Gramont and Haller, 2008).

5-Fluorouracil toxicity is modulated by FA and interferon-α (IFN*α*) (Corfu-A Study Group, 1995; Van Triest *et al*, 2000). Among several other mechanisms, FA increases the concentration of the cofactor 5,10-methylenetetrahydrofolate, thereby stabilising the ternary complex formation of 5-fluoro-2′-deoxyuridine-5′-monophosphate, with thymidylate synthase inhibiting DNA synthesis (Van Triest *et al*, 2000), whereas INF*α* enhances 5-FU metabolism and, moreover, has immunomodulating and antiangiogenic effects (Makower and Wadler, 1999; Slaton *et al*, 1999). The aim of this trial was to improve adjuvant chemoradiotherapy of rectal cancer by modulating 5-FU with either FA or IFN*α*. Secondary aims were to characterise toxicity of the regimens and identify clinical and pathological parameters influencing recurrence and prognosis.

## Patients and methods

### Ethics

The German ‘Research Group Oncology of Gastrointestinal Tumors’ (FOGT) designed a prospective randomised trial (FOGT-2) to optimise adjuvant treatment of rectal cancer conform to GCP/ICH rules and respecting the Helsinki Declaration (1989) to improve adjuvant treatment of locally advanced rectal cancer. It was approved by the Ethics Committee of the University of Ulm No. 87/91) and supervised by an independent study monitor. A similarly designed trial (FOGT-1) was performed in colon cancer (Link *et al*, 2005).

### Patient eligibility criteria

Patients had a medical history, physical examination, ECG, colonoscopy, complete blood cell count, and chemistry, including liver and renal function parameters and carcinoembryonic antigen. Distant metastases were excluded by abdominal ultrasound, chest X-ray, and intraoperative liver palpation. Computed tomography or MRI scans were optional.

Eligibility was defined as potentially curative *en bloc* resection (*R*_0_) of an adenocarcinoma of the rectum with a lower tumour edge within 12 cm from the anal verge determined by rectoscopy, a pathological UICC stage II (pT3/4pN0M0) or III (pT1-4pNposM0) with examination of at least 12 lymph nodes, a white blood count ⩾3500 *μ*l^−1^, a platelet count ⩾100 000 *μ*l^−1^, a ECOG performance status of 0 or 1, and written informed consent. Ineligible were patients not fulfilling these criteria or having a history of cancer, except for adequately treated superficial basal or squamous cell skin cancer or *in situ* carcinoma of the cervix, getting previous radio- or chemotherapy, pregnant or nursing women, others having severe concomitant diseases limiting life expectancy or not allowing chemotherapy, and with social conditions not allowing a 5-year follow-up.

### Surgical procedures

Anterior resections (AR) including Hartmann procedures and abdominoperineal resections (APR) had to be performed according to the recommendations of the German Cancer Society (Herfahrt and Schlag, 1991). A distal free resection margin of 3 cm was required for ARs and a wide resection of the levators close to the pelvis wall in case of APRs.

### Pathological evaluation

The fourth version of the UICC/TNM classification was used to document the pathological staging. Results in this paper are reported according to the sixth version. Overall, 57 patients initially documented as pN3 (central positive lymph nodes, fourth version) were summarised with the group of pN2. *R*_0_ was defined as complete resection to all directions without limit (0 mm). CRM was not recorded. No central pathological review was performed.

### Stratification and randomisation procedures

Randomisation was performed during a phone call according to an allocation sequence generated by the Institute of Biometrics of the University of Ulm. Patients were stratified according to the centre, pT (pT1/2 *vs* pT3/4), and lymph node status (pN0 *vs* pN1 *vs* pN2).

### Chemotherapy

At the time of the trial design, systemic adjuvant therapy of rectal cancer was carried out analogous to the recommended standard in colon cancer, consisting of 5-FU and oral levamisol for 12 months (NIH Consensus Conference, 1990). Therapy was scheduled to begin 14 days after surgery. All patients received 5-FU and levamisol. Levamisol (50 mg) was given orally three times on 3 consecutive days every 2 weeks (days 1–3). 5-Fluorouracil (450 mg m^−2^) was administered as infusion for 60–120 min on days 1–5. At 28 days after this loading course, 5-FU was given once weekly for 48 weeks and, if tolerated well, increased to 500 mg m^−2^. During irradiation, 5-FU was reduced to 80%. Folinic acid (200 mg m^−2^, Rescuvolin, Medac GmbH, Hamburg, Germany) was given as a short infusion (10 min) before 5-FU. Interferon-*α* (Roferon, Roche, Grenzach-Wyhlen, Germany) treatment consisted of 6 × 10^6^ IU as subcutaneous self-injection 3 × weekly. Training of self-injection was initiated on day 28.

### Radiation

Radiotherapy consisted of 50.4 Gy (45 Gy with 5.4 Gy small volume boost) delivered in fractions of 1.8 Gy 5 × weekly starting 6–8 weeks after surgery and was carried out lying face down and using a three-field technique. The target volume included the primary tumour and its mesentery with vascular supply containing the peri-rectal, pre-sacral, and internal iliac nodes. The upper limit was the L5/S1 junction, the dorsal limit the outer face of the sacrum/coccygis, the ventral limit the inner bone of the os pubis, and the lower limit at least 3 cm below the anastomosis in case of AR, and including the perineum in case of APR.

### Toxicity

Toxicity was evaluated according to the WHO criteria. Follow-up during adjuvant treatment as well as dose-reduction procedures in case of grade III or IV toxicities were described (Link *et al*, 2005). Severe toxicities were reported to the German drug authority ‘BfArM’.

### Follow-up

Follow-up was performed 4-monthly for 2 years and 6-monthly for 3 years, including history, physical examination, white blood count, liver and renal function, and carcinoembryonic antigen. Computed tomography of the pelvis, abdominal ultrasound, and chest X-ray were performed annually, and colonoscopy biannually. Additional annual follow-up exceeding 5 years was optional.

### Statistical analysis and end points

The primary objective was to improve adjuvant 5-FU chemoradiotherapy. Our hypothesis was that modulation of 5-FU by addition of either FA or INF*α* may increase overall survival (OS).

For sample size estimation, the following assumptions were made: the 5-year OS rate of 5-FU was estimated to be 58% (Krook *et al*, 1991). If the 5-year OS rate for one of the additives is 10% points higher compared with 5-FU, the study has 80% power to detect superiority at a level of significance of 5% (one-sided), with a sample size of 280 subjects per group. 5-Fluorouracil alone was compared with 5-FU with the addition of FA and INF*α*. Owing to the fact that the INF*α* arm was closed in 1999 (see Results), a confirmatory comparison was only carried out for 5-FU alone *vs* 5-FU+FA (log-rank test).

Primary end point of the study was OS. Overall survival was compared by log-rank testing for 5-FU alone and 5-FU+FA. Secondary end points were recurrence-free survival (RFS), LR, toxicity, and treatment compliance. Overall survival was computed from the start of chemotherapy until death of any cause (events) or until the last observation date (censored observations). Recurrence-free survival was defined as time from the start of chemotherapy until diagnosis of any tumour recurrence or tumour-related death (events) or until death due to other reasons or last observation date (censored observations). Local recurrence was defined as time from the start of chemotherapy to diagnosis of local tumour recurrence (events) or death, last observation date, or sole occurrence of distant metastases (censored observations). Survival curves were generated by the Kaplan–Meier method. Five-year survival rates are shown in % with 95% confidence intervals. Toxicity rates were compared between the treatment arms using the *χ*^2^ test. Stratified Kaplan–Meier analyses were performed to detect variables influencing LR, RFS, and OS, and compared with the log-rank test. All these tests were used for exploratory data analysis. Statistical analysis was performed using SAS version 9.1 (SAS Institute Inc., Carry, NC, USA).

## Results

### Patient and tumour characteristics

**Figure 1:**
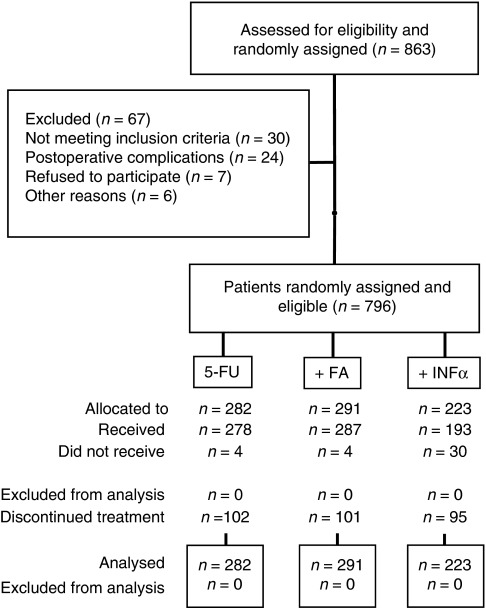
CONSORT diagram.

A total of 863 patients from 55 hospitals were enrolled. Of these, 67 (7.8%) were regarded as drop-outs (Figure 1). Clinical and pathological characteristics of the remaining 796 patients are summarised in Table 1.

### Adjuvant treatment and compliance

Treatment was started on 29 July 1992 for the first patient and finished on 6 March 2003 for the last patient. All 796 patients received 5-FU chemotherapy (Figure 1). Four patients randomised to 5-FU alone received additional FA and four randomised to 5-FU+FA received only 5-FU (Figure 1). Self-injection of INF*α* was refused by 30 patients (Figure 1). In total, 11 received the 5-FU loading course and discontinued any further adjuvant therapy. In all, 19 continued adjuvant treatment without INF*α*, of these seven received 5-FU alone and 12 asked to receive 5-FU+FA.

The administration of the complete 12-month course of adjuvant chemoradiotherapy was documented for 50.3% (400 out of 796) of the patients, 50.4% (142 out of 282), 53.3% (155 out of 291), and 46.2% (103 out of 223) of the 5-FU, 5-FU+FA, and 5-FU+INF*α* group, respectively (Figure 1, Table 2). At least 6 months were given to 67.7% (539 out of 796). Discontinuation was observed in 10.8% (*n*=86) within the first, 9.5% (*n*=76) within the second, 6.7% (*n*=53) within the third, and 10.8% (*n*=86) within the fourth quarter. No data about the duration of chemotherapy were available for 95 patients. Reasons for discontinuation of chemotherapy are shown in Table 2.

For patients discontinuing chemotherapy within the first quarter of treatment (*n*=86), radiation was not administered in 21 patients and was discontinued in six patients, whereas no data on radiation were available in 27 patients.

### Toxicity

Toxicity data were available for 685 patients (86%). World Health Organisation III and IV toxicities occurred in 37.5% (257 out of 685) of the patients. Despite the fact that 30 of 223 patients (13.5%) never received INF*α*, toxicities occurred in more patients (58.1%) receiving 5-FU+INF*α* than 5-FU (31.5%) and 5-FU+FA (27.7%) attributable to more frequent haematological, gastrointestinal, and neurological courses (*P*<0.001; Table 3). Toxicity-related abruption (23 out of 223, 10.3%) was also higher in comparison to 5-FU (14 out of 282, 5.0%) and 5-FU+FA (nine out of 291, 3.1%). This prompted the study review committee to close the INF*α* arm in February 1999.

Three deaths due to treatment-related toxicity were documented. One patient receiving 5-FU immediately died after the loading course owing to severe febrile neutropenia followed by pneumonia. Another patient receiving 5-FU+INF*α* died in month 7 of treatment owing to diarrhoea with massive dehydration and renal failure. The third patient (5-FU) died 6 months after completing chemoradiotherapy owing to infectious complications caused by fistulas in the pelvis without evidence of LR.

### Tumour recurrence

The median follow-up was 4.9 years (range: 0.0–16.7 years). In all, 349 recurrences have been reported resulting in a recurrence rate of 43.8% (Table 4). Recurrence was reported in seven patients after 5 years of follow-up.

**Figure 2:**
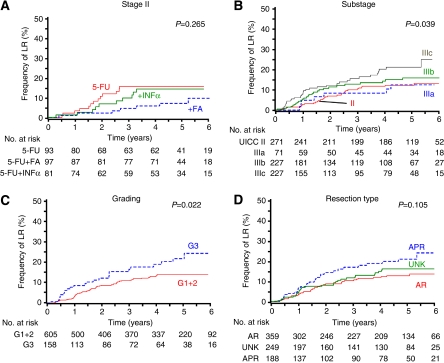
Cumulative frequency of local recurrence (LR): (**A**) LR in UICC stage II (pT3/4pN0) according to treatment; (**B**) LR according to UICC stage II (pT3/4pN0), and substages IIIa (pT1/2pN1), IIIb (pT3/4pN1), and IIIc (pT1-4pN2); (**C**) LR according to tumour grading; and (**D**) LR according to the type of resection: anterior resection (AR), abdominoperineal resection (APR), and resection type unknown (UNK). AR included nine Hartmann procedures.

Local recurrence was reported for 100 patients (12.6%), of which 45 patients of this group had both local and distant relapse. Treatment did not influence LR in stage III. In contrast, addition of FA reduced 5-year LR rate by 55% in stage II disease compared with 5-FU (Table 5). In stage II, IIIa, IIIb, and IIIc, 11.4% (31 out of 271), 11.3% (8 out of 71), 11.9% (27 out of 227), and 15.0% (34 out of 227) had LR, respectively. Patients with grading 1+2 and 3 had LR in 11.2% (68 out of 605) and 15.8% (25 out of 158), respectively, and patients undergoing AR and APR in 11.4% (41 out of 359) and 15.4% (29 out of 188), respectively. The cumulative frequency of LR with respect to adjuvant treatment in UICC stage II, UICC substage, grading, and resection type are summarised in Table 5 and plotted in Figure 2.

**Figure 3:**
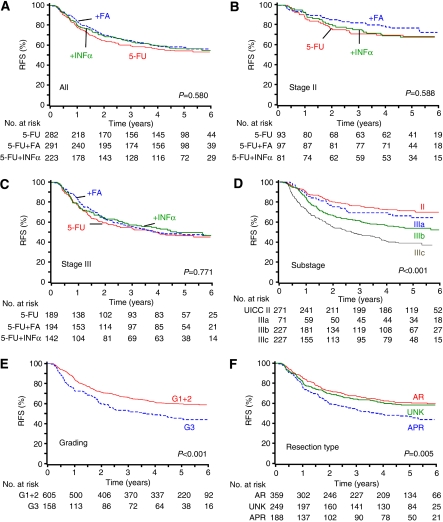
Recurrence-free survival according to: (**A**) treatment for all stages; (**B**) treatment of stage II (pT3/4pN0); (**C**) treatment of stage III (pT1-4pNpos); (**D**) UICC stage II (pT3/4pN0), and substages IIIa (pT1/2pN1), IIIb (pT3/4pN1), and IIIc (pT1-4pN2); (**E**) tumour grading (G1+2 *vs* G3); and (**F**) type of resection: anterior resection (AR), abdominoperineal resection (APR), and resection type unknown (UNK). AR included nine Hartmann procedures.

Distant metastases were reported in 284 patients (35.7%). The addition of FA tended to increase 5-year RFS in stage II, but not in stage III disease (Table 5). Recurrence-free survival was associated with UICC substage, tumour grading, and resection type (Table 5). Kaplan–Meier curves of RFS are shown in Figure 3.

### Survival

As of November 2009, 335 patients (42.1%) died, 43.3% of the patients (122 out of 282) receiving 5-FU, 40.9% of the patients (119 out of 291) receiving 5-FU+FA, and 42.2% of the patients (94 out of 223) receiving 5-FU+INF*α*. Disease-specific (disease-related) deaths occurred in 36.2% of the patients with 5-FU (102 out of 282), in 33.7% of the patients with 5-FU+FA (98 out of 291), and in 33.6% of the patients receiving with 5-FU+INF*α* (75 out of 223), combining to a total disease-specific death rate of 82.1% (275 out of 335). A total of 43 patients (12.8%) died of other reasons, including the three patients with treatment-related toxicity, whereas the cause of death was unknown in 17 patients.

**Figure 4:**
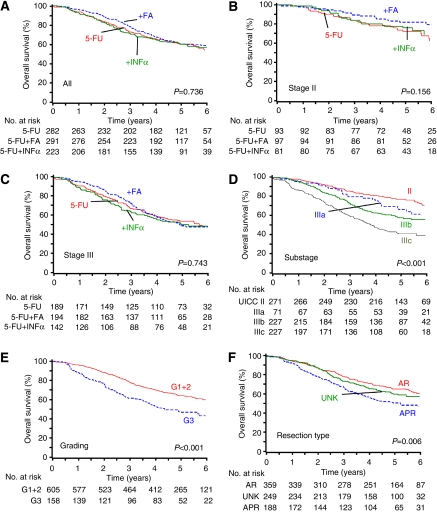
Overall survival according to: (**A**) treatment for all stages. 5-Fluorouracil *vs* 5-FU+FA: log-rank test, *P*=0.461; (**B**) treatment of stage II (pT3/4pN0); (**C**) treatment of stage III (pT1-4pNpos); (**D**) UICC stage II (pT3/4pN0), and substages IIIa (pT1/2pN1), IIIb (pT3/4pN1), and IIIc (pT1-4pN2); (**E**) tumour grading (G1+2 *vs* G3); and (**F**) type of resection: anterior resection (AR), abdominoperineal resection (APR), and resection type unknown (UNK). AR included nine Hartmann procedures.

5-Fluorouracil+FA tended to a superior OS rate after 3 years (78.3%) compared with 5-FU (72.8%) and 5-FU+INF*α* (70.9%). However, no differences were seen after 5 years (Table 5, Figure 4A). The addition of FA tended to an improved OS in stage II, whereas no effects were observed in stage III disease (Table 5, Figure 4B+C). Overall survival was influenced by UICC substage, tumour grading, and resection type (Table 5, Figure 4D–F).

## Discussion

Adjuvant chemoradiotherapy of locally advanced rectal cancer was established based on three trials, including 104 (Gastrointestinal Tumor Study Group, 1985), 204 (Krook *et al*, 1991), and 555 patients (Fisher *et al*, 1988). Our trial design was based on the results of these studies not allowing a ‘surgery-only’ arm. The main problem of the study was patient recruitment. Nevertheless, duration and time of recruitment are comparable to other European rectal cancer trials launched in the early 1990s (Sauer *et al*, 2004; Bosset *et al*, 2006; Gérard *et al*, 2006). The German ARO-CAO-AIO-94 study compared pre- *vs* postoperative chemoradiotherapy (Sauer *et al*, 2004), and the two French trials compared pre-operative radiotherapy with chemoradiotherapy with or without postoperative chemotherapy (Bosset *et al*, 2006; Gérard *et al*, 2006). Two trials initiated in the United States in the early 1990s comparing pre-operative chemoradiotherapy with standard, postoperative chemoradiotherapy by the RTOG (trial 94-01) and the National Surgical Adjuvant Breast and Bowel Project (protocol R-03) were closed prematurely owing to low enrolment (Hyams *et al*, 1997). Trials starting in the later 1990s compared pre-operative short-course radiotherapy *vs* TME surgery alone (Peeters *et al*, 2007) or *vs* postoperative selective chemoradiotherapy (Sebag-Montefiore *et al*, 2009) or pre-operative short-course radiotherapy *vs* chemoradiotherapy applying TME surgery (Bujko *et al*, 2006). With the exception of the Swedish Rectal Cancer Trial (1997), rectal cancer trials involving multimodal treatment revealed improvement of local control without benefit for prognosis (Sauer *et al*, 2004; Bosset *et al*, 2006; Bujko *et al*, 2006; Gérard *et al*, 2006; Peeters *et al*, 2007; Sebag-Montefiore *et al*, 2009).

In the area of TME surgery, prognosis of patients with locally advanced rectal cancer primarily depends on the occurrence of distant metastases. No study could show an improvement of prognosis in multimodal treatment of rectal cancer in comparison to standard 5-FU (de Gramont and Haller, 2008). Our study aimed to improve prognosis by modulating 5-FU by either addition of FA or INF*α*. In parallel, we carried out an equivalent study in colon cancer, except radiation including 855 patients (Link *et al*, 2005). Similar to our colon cancer study, INF*α* increased toxicity in rectal cancer, too, without survival benefit. The effectiveness of combining 5-FU with FA in colon cancer is generally accepted and was confirmed in our colon trial increasing the 5-year OS rate from 61 to 72% using the same drug administration and protocol design (Link *et al*, 2005). More than 90% of patients with stage III colon cancer were included. In this study, not a trend of benefit was observed in node-positive (stage III) rectal cancer. However, there seemed to be a benefit of FA addition in stage II cutting LR rate by half and enhancing OS by almost 10 points of percentage (82.1 *vs* 72.7%) compared with 5-FU. A pooled analysis of Scandinavian patients comparing surgery only with postoperative adjuvant 5-FU-based chemotherapy in rectal cancer showed a similar trend. Patients with stage II seemed to benefit, whereas there was no effect of adjuvant treatment compared with surgery alone in stage III (Glimelius *et al*, 2005). A subgroup analysis of EORTC Trial 22921 comparing pre-operative (chemo)radiotherapy with or without postoperative chemotherapy in a 2 × 2 factorial design revealed that responders (ypT0–2) seemed to benefit from adjuvant chemotherapy in contrast to non-responders (ypT3–4) (Collette *et al*, 2007). These observations suggest that especially non-metastasised and radiosensitive tumours may benefit from adjuvant 5-FU treatment with the addition of FA, whereas non-responding and lymph node-positive tumour may not.

On the basis of this observation in stage II of our study that the reduction of LR was associated with an improvement of RFS and OS and the ineffectiveness of FA in stage III, some assumptions can be made. First, the addition of FA (200 mg m^−2^) may enhance the effect of 5-FU as a radiosensitiser to improve local control. Second, the addition of FA may be ineffective to avoid recurrence at a stage of rectal cancer at which metastatic spread is already present in lymph nodes. Third, chemosensitivity of rectal cancer may differ from that of colon cancer. This is supported by comparisons with colon cancer trials (Glimelius *et al*, 2005; Link *et al*, 2005) and other trials failing to show an improvement of adjuvant 5-FU monotherapy in rectal cancer (QUASAR Collaborative Group, 2000; Tepper *et al*, 2002; Dahl *et al*, 2009). In addition, new combinations, which also showed effectiveness in colon cancer treatment, failed to show any benefit in rectal cancer so far (Glynne-Jones *et al*, 2010; Weiss *et al*, 2010). The German CAO/ARO/AIO-04 rectal cancer trial comparing standard 5-FU neoadjuvant and adjuvant treatment with an intensified protocol, including oxaliplatin in the pre- and postoperative setting (Rödel and Sauer, 2007), was recently closed for recruitment. However, no differences in the rate of pathological complete response to neoadjuvant therapy as a surrogate marker for overall prognosis were reported so far. The effects on distant metastasis and final outcome render evaluation after sufficient follow-up time in a few years for this and other ongoing European and United States trials.

The duration of adjuvant chemotherapy in our trial was 12 months. All patients received oral levamisol. Presently, a duration of 6–8 months is recommended with omission of levamisol. These recommendations are mainly based on results obtained from colon cancer trials showing no difference in outcome omitting levamisol and shortening the duration of chemotherapy. The four-arm INT-0089 trial, including patients with high-risk stage II and stage III colon cancer, revealed no significant difference between adjuvant therapy with 5-FU+FA (low dose, 20 mg m^−2^ or high dose, 500 mg m^−2^) for 7–8 months and the 12-month 5-FU+LEV standard and an increase in the 5-year OS rate from 63% (12-month 5-FU+LEV) to 67% for 7–8 months 5-FU+LEV+FA (low dose) (Haller *et al*, 1998). The NCCTG/NCIC trial (O’Connell *et al*, 1998), including 915 similarly staged patients, displayed 5-year OS rates of 64% for 12-month 5-FU+LEV, of 61% for 12-month 5-FU+LEV+FA, of 69% for 6-month treatment with 5-FU+FA, and of 59% for 6-month treatment with 5-FU+LEV. Except the two 6-month treatment arms, the differences between the treatment arms were not significant. On the basis of the results, a 6- to 8-month adjuvant treatment seemed to be equivalent to a 12-month 5-FU+LEV treatment after the addition of FA. Furthermore, omission of LEV seemed to be justified without compromising the survival benefit in colon cancer.

Attention has be drawn to a variety of additional anatomical and surgical factors influencing the outcome of rectal cancer. The tumour distance from the anal verge seems of great importance as shown in our study as well. As a result, patients with low rectal cancer undergoing an APR had a 63% higher LR rate than patients undergoing an AR in our and other studies (Wibe *et al*, 2002; den Dulk *et al*, 2009). Localisation of the tumour in the rectum may be another essential prognostic factor. To achieve a complete resection with negative circumferential resection margins, it is important that the tumour is covered with mesorectal fatty tissue (Heald *et al*, 2004; Nagtegaal and Quirke, 2008). The mesorectum is thinned out in the lower parts, especially in the front (Heald *et al*, 2004). Moreover, the individual surgeon may be also another important prognosticator (Martling *et al*, 2000). All these factors and the combination with radiotherapy may dilute the positive effect of 5-FU modulation by FA in rectal cancer, which seems so obvious in colon cancer.

In summary, we could not show a benefit of modulating 5-FU with either FA or INF*α* in adjuvant chemoradiotherapy of locally advanced rectal cancer despite a tendency in improved 3-year survival. Nevertheless, our results point out to a potential long-term benefit of FA in stage II disease. Therefore, in our opinion, this protocol can be recommended for adjuvant chemoradiotherapy of stage II disease. Owing to a reduction in LR, we conclude that this effect may be due to increased efficacy of chemoradiotherapy. In the future, this protocol may be recommended for patients not having received neoadjuvant treatment being diagnosed with a pT3c/dpN0 tumour or with a small CRM (<2 mm). Patients with pT3a/bpN0 or a large CRM (>2 mm) may undergo observation. The effect of adjuvant treatment, even 5-FU monotherapy, after neoadjuvant chemoradiotherapy and high quality of surgery renders re-evaluation.

Our study further confirmed important prognostic factors like grading, type of resection, and UICC substage (Gunderson *et al*, 2002). In view of the efficacy of our FA protocol in colon cancer, we further conclude that rectal cancer may be a separate entity with different chemosensitivity. This is supported by numerous observations of differences in genetic alterations or target gene expression like microsatellite instability or expression of thymidylate synthase (Kornmann *et al*, 2003; Allen and Johnston, 2005; Wilson *et al*, 2007).

As all attempts to optimise and develop new combinations for chemotherapy of rectal cancer have failed so far and no really promising additional multimodal treatment options are under evaluation at present, it seems important to focus on approaches minimising over-treatment. For example, accurate pre-therapeutic MRI-based local staging may better identify patients that can profit from neoadjuvant treatment based on the CRM. In conjunction with additional individual prognostic markers like grading, tumour location, and tumour substaging, this may help to reduce the need for multimodal strategies. Future trials should therefore aim at optimising available multimodal options for high-risk subgroups, thereby reducing the overall number of patients undergoing multimodal treatment. A recent survey asking laypersons about their preferred treatment choices further would support this strategy (Kornmann *et al*, 2008). This may save toxicity and increase quality of life without hampering prognosis. These efforts may eventually help to individualise and optimise multimodal treatment of locally advanced rectal cancer in the future.

## Figures and Tables

**Table 1:** Clinical and pathological characteristics

	**Treatment**			
**Patients Number**	**5-FU (*N*=282)**	**5-FU+FA (*N*=291)**	**5-FU+IFN*α* (*N*=223)**	**Total (*N*=796)**
*Age (years)*				
Median	61.6	61.4	61.2	61.4
Range	31.5–81.4	23.0–81.4	29.6–86.3	23.0–86.3
				
*Sex*				
Male	180	191	140	511
Female	102	100	83	285
				
*Type of resection* a				
AR^b^	135	126	98	359
APR	60	78	50	188
Unknown	87	87	75	249
				
*UICC stage*				
II	93	97	81	271
A T3 N0	85	89	71	245
B T4 N0	8	8	10	26
III	189	194	142	525
A T1/2 N1	26	25	20	71
B T3/4 N1	76	87	64	227
C T1–4 N2	87	82	58	227
				
*Tumour depth (T)*				
1	3	2	4	9
2	33	35	18	86
3	225	227	182	634
4	21	27	19	67
				
*Lymph nodes (N)*				
0	93	97	81	271
1	102	112	84	298
2	87	82	58	227
				
*Grading (G)*				
1+2	212	219	174	605
3	55	61	42	158
Unknown	15	11	7	33

Abbreviations: AR=anterior resections; APR=abdominoperineal resections; FA=folinic acid; 5-FU=5-fluorouracil; IFN*α*=interferon-*α*; UICC=International Union Against Cancer.

a Type of resection was determined retrospectively.

b Including Hartmann procedures (5-FU, *n*=3; 5-FU+FA, *n*=3; 5-FU+INF*α*, *n*=3; total, *n*=9).

**Table 2:** Reasons for treatment discontinuation

	**Treatment**			
	**5-FU (*N*=282)**	**5-FU+FA (*N*=291)**	**5-FU+IFN*α* (*N*=223)**	**Total (*N*=796)**
Patient's demand	33	45	32	110
Toxicity	14	9	23	46
Disease progression	45	34	29	108
Secondary tumour	2	1	1	4
Death	3	3	2	8
Other reasons	3	4	6	13
Missing information	2	5	2	9
Total (in %)	102 (36)	101 (35)	95 (43)	298 (37)

Abbreviations: FA=folinic acid; 5-FU=5-fluorouracil; IFN*α*=interferon-*α*.

**Table 3:** Toxicities WHO III+IV

	**Treatment**			
	**5-FU (*N*=282)**	**5-FU+FA (*N*=291)**	**5-FU+INF*α* (*N*=223)**	**Total (*N*=796)**
*Toxicity data available* a	*N*=241	*N*=253	*N*=191	*N*=685
Haematological^b^	13	6	48	67
Nausea/vomiting	7	12	20	39
Diarrhoea	38	41	53	132
Fever	1	3	13	17
Skin	18	16	23	57
Neurological	4	6	13	23
Others^c^	12	19	20	51
No cause stated	73	67	107	247
				
*Caused by*				
Chemotherapy	35	31	56	122
Radiotherapy	19	22	7	48
Both	22	17	48	87
Number of patients^d^ (in %)	76 (32)	70 (28)	111 (58)	257 (38)

Abbreviations: FA=folinic acid; 5-FU=5-fluorouracil; IFN*α*=interferon-*α*; WHO=World Health Organisation.

a The results of toxicity were based on the analysis of 685 patients for whom toxicity data were available.

b Number of documented toxicities >WHO II.

c Including obstipation and infections, as well as renal, pulmonal, and cardiac toxicity.

d Total number of patients affected by any toxicity >WHO II.

**Table 4:** Localisation and frequency of tumour recurrence

	**Treatment**			
	**5-FU (*N*=282)**	**5-FU+FA (*N*=291)**	**5-FU+INF*α* (*N*=223)**	**Total (*N*=796)**
*Total number of patients*	129	123	97	349
Recurrence rate (in %)	45.7	42.3	43.5	43.8
Local recurrence (only) (in %)	21 (7.4)	16 (5.5)	18 (8.1)	55 (6.9)
Local and distant recurrence (in %)	18 (6.4)	15 (5.2)	12 (5.4)	45 (5.7)
Distant recurrence (only) (in %)	88 (31.2)	88 (30.2)	63 (28.3)	239 (30.0)
Unknown localisation (in %)	2 (0.7)	4 (1.4)	4 (1.8)	10 (1.3)
				
*Distant metastases (events)* a				
Liver	60	54	42	156
Lung	41	35	25	101
Peritoneum	16	8	8	32
Bone	2	11	4	17
Other locations	23	22	17	62

Abbreviations: FA=folinic acid; 5-FU=5-fluorouracil; IFN*α*=interferon-α.

a Owing to the fact that some patients showed more than one location of distant metastases, the total number of distant metastases is higher than the patient number.

**Table 5:** Five-year rates of LR, RFS and OS by risk group

		**Five-year rates**		
**Risk groups**	**No.**	**LR in % (95% CI)**	**RFS in % (95% CI)**	**OS in % (95% CI)**
*Treatment*				
5-FU	282	16.7 (12.3–22.5)	54.4 (48.2–60.1)	60.3 (54.3–65.8)
5-FU+FA	291	13.6 (9.6–19.0)	58.0 (51.9–63.6)	60.4 (54.4–65.8)
5-FU+INF*α*	223	17.1 (12.2–23.8)	56.5 (49.5–63.0)	59.9 (53.0–66.1)
				
*Treatment of UICC II*				
5-FU	93	16.1 (9.6–26.1)	68.4 (57.8–76.8)	72.7 (62.3–80.6)
5-FU+FA	97	7.2 (3.3–15.5)	76.7 (66.8–84.0)	82.1 (72.8–88.5)
5-FU+INF*α*	81	14.6 (8.1–25.5)	67.3 (55.8–76.5)	76.1 (65.1–84.0)
				
*Treatment of UICC III*				
5-FU	189	17.0 (11.5–24.7)	47.2 (39.6–54.3)	54.2 (46.7–61.1)
5-FU+FA	194	17.5 (11.9–25.2)	48.1 (40.6–55.2)	49.4 (42.0–56.4)
5-FU+INF*α*	142	18.7 (12.2–28.1)	49.8 (40.8–58.2)	50.5 (41.8–58.6)
				
*UICC stage*				
II (pT3–4 pN0)	271	12.4 (8.8–17.4)	71.0 (65.1–76.1)	77.1 (71.5–81.7)
IIIa (pT1–2 pN1)	71	12.6 (6.2–24.8)	64.0 (51.2–74.3)	66.5 (54.0–76.3)
IIIb (pT3–4 pN1)	227	16.1 (11.2–22.7)	53.2 (46.1–59.7)	57.6 (50.7–63.9)
IIIc (pT1–4 pN2)	227	21.4 (15.4–29.3)	38.3 (31.7–44.8)	40.7 (34.2–47.1)
				
*Tumour grading*				
G1+2	605	14.1 (11.2–17.6)	59.2 (55.0–63.1)	63.9 (59.8–67.6)
G3	158	21.9 (15.0–31.4)	45.0 (36.7–53.0)	46.7 (38.6–54.3)
				
*Type of resection*				
AR	359	12.9 (9.5–17.4)	60.4 (55.0–65.4)	65.3 (60.1–70.1)
APR	188	21.1 (14.9–29.3)	46.2 (38.6–53.5)	50.8 (43.3–57.8)
Unknown	249	16.2 (11.6–22.5)	57.8 (51.1–63.8)	59.8 (53.4–65.7)

Abbreviations: AR=anterior resections, including Hartmann procedures (*n*=9; APR=abdominoperineal resections; CI=confidence interval; FA=folinic acid; 5-FU=5-fluorouracil; IFN*α*=interferon-*α*; LR=local recurrence; OS=overall survival; RFS=recurrence-free survival; UICC=International Union Against Cancer.

## Appendix

The following investigators participated in the trial and recruited at least 10 patients: E Kettner, Department of Medicine, Städtisches Klinikum, Magdeburg; H Schramm, Department of Surgery, Waldklinikum, Gera; W Baumann, Department of Surgery, Klinik am Eichert, Göppingen; T Liersch, Department of Surgical Oncology, University of Göttingen; K Ridwelski, Department of Surgery, University of Magdeburg, Magdeburg (current address: Department of Surgery, Klinikum Dessau); J Gabriel, Department of General and Transplantation Surgery, University of Rostock; K-U Zerbian, Department of Surgery, Kreiskrankenhaus Lüdenscheid; KH Ebert, Department of Surgery, St Martinus-Hospital, Olpe; H Bewersdorf, Department of Medicine I, Ostalbklinikum, Aalen; H-F Weiser, Department of Surgery, Ev.-Luth. Diakoniekrankenhaus Rotenburg; U Schenker, Department of Surgery, Park-Krankenhaus, Leipzig; B Karn, Department of Surgery, Klinikum Bad Salzungen; W Oettinger, Department of Surgery, and W Weber, Department of Medicine, Krankenhaus der Barmherzigen Brüder, Trier; F Schwanghart, Department of Medicine, St Elisabeth Krankenhaus, Bad Kissingen; P Mattes, Department of General Surgery, Städt. Krankenanstalten Esslingen; K Fleischer, Department of Medicine, Helfensteinklinik, Geislingen; J Kuhlgatz, Department of Surgery, Klinikum Fulda (current address: Department of Surgery, Albert-Schweitzer-Krankenhaus Northeim); N Heni, Department of Medicine, D Höfer, Department of Surgery, Kreiskrankenhaus Biberach; G Kraatz, Department of Medicine, University of Greifswald; J Vogt, Department of Surgery, St Vinzenz-Krankenhaus Hanau; V Mendel, Department of Surgery, Diakonissenkrankenhaus Flensburg; G Simonis, Department of Surgery, Krankenhaus d. Bundesknappschaft, Püttlingen; J Albrecht, Department of Surgery, Kreiskrankenhaus Schorndorf; M Anlauf, Department of Medicine, Zentralkrankenhaus Reinkenheide, Bremerhaven; E-U Steinhauer, Department of Hematology and Oncology, Städt. Kliniken Kassel; and J Limmer, Department of Surgery, Winterberg-Kliniken, Saarbrücken.
